# Supplementary material for: Vitamin D-related polymorphisms and vitamin D levels as risk biomarkers of COVID-19 disease severity
Source: Sci Rep. 2021 Oct 21;11:20837. doi: 10.1038/s41598-021-99952-z (PMC8531279; doi:10.1038/s41598-021-99952-z)
Supplement: Supplementary file 1 — Supplementary Information. [file 41598_2021_99952_MOESM1_ESM.pdf]

Vitamin D-related polymorphisms and vitamin D levels as risk biomarkers of COVID-19 disease severity

Ana Teresa Freitas [2,3], Conceição Calhau [4,5], Gonçalo Antunes [2], Beatriz Araújo [11], Matilde Bandeira [6,15], Sofia Barreira [6,15], Filipa Bazenga [11], Sandra Braz [7], Daniel Caldeira [1,10], Susana Constantino Rosa Santos [1], Ana Faria [4], Daniel Faria [13,3], Marta Fraga [9], Beatriz Nogueira-Garcia [10], Lúcia Gonçalves [2], Pavlo Kovalchuk [2], Luísa Lacerda [11], Hugo Lopes [2], Daniel Luís [2], Fábio Medeiros [8], Ana M. P. Melo [13,14], José Melo-Cristino [9], Ana Miranda [9], Clara Pereira [2], Ana Teresa Pinto [1], João Pinto [11], Helena Proença [9], Angélica Ramos [11,12], João P. R. Rato [13,14], Filipe Rocha [1] , Júlio César Rocha [4,5], André Moreira-Rosário [4,5], Helena Vazão [2], Yuliya Volovetska [9], João-Tiago Guimarães [11,12], Fausto Pinto [1,10*]

1 Centro Cardiovascular da Universidade de Lisboa (CCUL), CAML, Faculdade de Medicina, Universidade de Lisboa, Lisboa, Portugal;
2 HeartGenetics, Genetics and Biotechnology SA, Biocant Park, Portugal;
3 INESC-ID, Instituto Superior Técnico, University of Lisbon, Lisboa, Portugal;
4 Nutrition and Metabolism, NOVA Medical School, Universidade Nova de Lisboa, Lisboa, Portugal;
5 CINTESIS - Center for Health Technology and Services Research, Portugal;
6 Rheumatology Department, Hospital de Santa Maria, Centro Hospitalar Universitário Lisboa Norte, Centro Académico de Medicina de Lisboa, Lisboa, Portugal;
7 Internal Medicine Department, Santa Maria Hospital, Centro Hospitalar Universitário Lisboa Norte, Faculdade de Medicina da Universidade de Lisboa, Portugal;
8 Infectious Disease Department, Santa Maria Hospital - CHULN, Lisboa, Portugal;
9 Clinical Pathology Department, Hospital Santa Maria, Centro Hospitalar Universitário Lisboa Norte, EPE, Lisbon, Portugal;
10 Cardiology Department, Hospital Universitário de Santa Maria - CHULN, Lisboa, Portugal;
11 Clinical Pathology Department, Centro Hospitalar Universitário de São João, Portugal;
12 Biomedicine Department, Faculdade de Medicina, EPIUnit, Instituto de Saúde Pública, Universidade do Porto, Portugal;
13 Portuguese Infrastructure of Biological Data – BioData.pt, Portugal;
14 Instituto Gulbenkian de Ciência, Oeiras, Portugal;
15 Rheumatology Research Unit, Instituto de Medicina Molecular, Faculdade de Medicina, Universidade de Lisboa, Centro Académico de Medicina de Lisboa, Lisboa, Portugal.

Supplemental data

Demographic, clinical and phenotypic characteristics

For the data analysis, a total of 491 patients with a laboratory confirmed positive COVID-19 test and completed data registries, 371 (75.6%) from Santa Maria hospital and 120 (24.4%) from São João hospital, were considered. There were 217 female and 266 male patients with COVID-19 with mean ± SD age of 69.7±15.8 years. Dead, severe and moderate disease were observed in 18.5%, 21.8% and 59.7% of patients, respectively. Demographic and clinical characteristics of these patients are reported in Table i below.

The majority of individuals had three or more pre-existing comorbidities, with hypertension (63.1%), diabetes (31.8%) and obesity (23.4%) diseases being the most frequent ones. Figure i presents the frequency of the comorbidities and Figure ii presents the distribution of the number of significant comorbidities.

During hospitalization, most patients needed oxygen supply (76.4%), and 22.2 percent were admitted to the ICU due to the necessity of non-invasive or invasive mechanical ventilation as determined by the health care providers. The mean length of stay was 17.9±16.7 days, and 91 patients died.

In COVID-19 positive patients, the prevalence of vitamin D deficiency was 61.7% and 68.3% in Santa Maria and São João hospitals, respectively (Figure iii), using the Endocrine Society cutoff (Figure iv). On Chi square test, the differences in the prevalence of vitamin D deficiency (vitamin D level < 20 ng/mL), insufficient (vitamin D level [20, 30[ng/mL) and sufficient (vitamin D level >= 30 ng/mL), among the two hospitals, were 6.6%, 11.4% and 3.9%, respectively. These differences are statistically significant with a p-value of 0.04. The analysis related with the Vitamin D levels was also performed for each hospital's dataset separately. It was observed that the

correlation results pointed in the same direction in both subsets and gained statistical significance when they were combined in a single dataset (when compared with each independent result). The reduced sample size of each hospital's subset leads to less statistical power in the results, particularly in São João's dataset that is smaller. Considering these observations, the combined dataset was chosen to be the reference for the presented results.

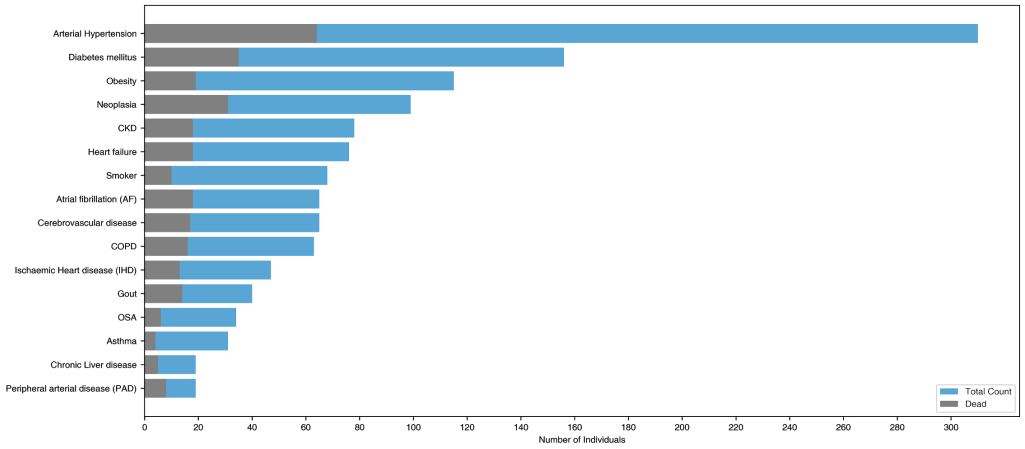

Figure i: Frequency of comorbidities

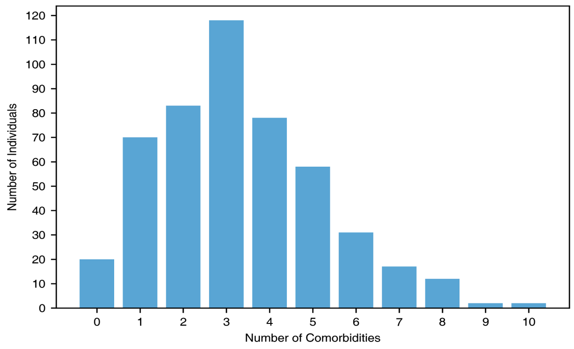

Figure ii: Distribution of the number of significant comorbidities.

From a total of 311 patients with vitamin D deficiency, 68 died, 69 had a severe response and 174 had a moderate response to COVID-19.

Table i: Demographic and clinical characteristics of the patients

		#	%
Patients		491	100.0
Age		69.7±15.8	-
Sex	Male	266	54.2
	Female	217	44.2
	n.a.	8	1.6
COVID-19 severity (WHO clinical progression scale - Table ii)	4	110	22.4
	5	183	37.3
	6	77	15.7
	7	6	1.2
	8	12	2.4
	9	12	2.4
	10	91	18.5
Vitamin D levels	deficient	311	63.3
	insufficient	120	24.4
	sufficient	59	12.0
Comorbidities	Arterial hypertension	310	63.1
	Diabetes mellitus	156	31.8
	Obesity	115	23.4
	Neoplasia	99	20.2
	CKD	78	15.9
	Heart failure	76	15.5
	Smoker	68	13.8
	Atrial fibrillation (AF)	65	13.2
	Cerebrovascular disease	65	13.2
	COPD	63	12.8

	Ischaemic heart disease (IHD)	47	9.6
	Gout	40	8.1
	OSA	34	6.9
	Asthma	31	6.3
	Chronic liver disease	19	3.9
	Peripheral arterial disease	19	3.9
Drugs	Statins	184	37.5
	Diuretic	166	33.8
	Beta-blocker	133	27.1
	Calcium channel blockers	125	25.5
	ARA	121	24.6
	Anti-Aggregate	104	21.2
	ACE inhibitors	96	19.6
	Metformin	93	18.9
	Anticoagulant	68	13.8
	DPP-4 inhibitors	49	10.0
	Corticosteroids	46	9.4
	Insulin	38	7.7
	Vitamin D medication	33	6.7
	Non-steroidal anti-inflammatory	28	5.7
	ADO - others	26	5.3
	Immunosuppressant - others	24	4.9
	Spironolactone	20	4.1
	iSGLT2/aGLP1	20	4.1
	Antiarrhythmics	16	3.3

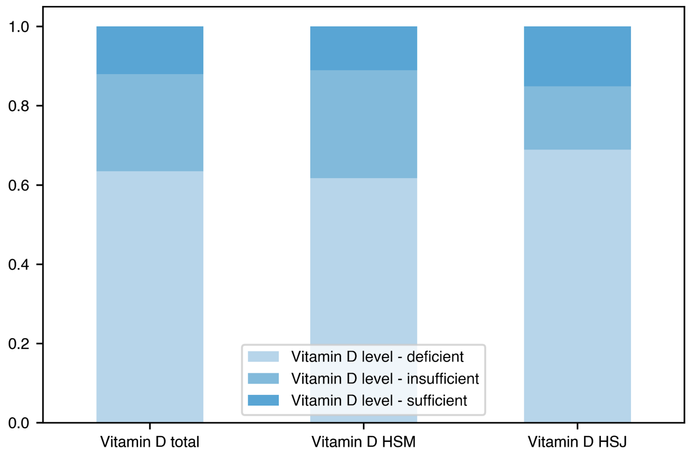

Figure iii: Vitamin D levels (categories %) in all recruited patients and by hospital distribution. (Deficient: < 20 ng/mL; Insufficient: [20, 30[ng/mL; Sufficient: >= 30 ng/mL).

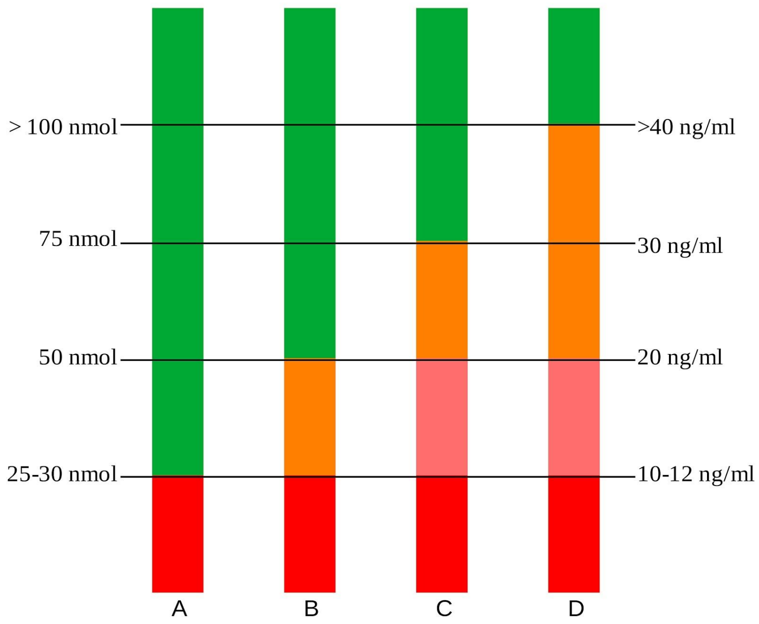

Figure iv: Interpretation of serum values of 25 (OH)D according to different agencies and countries. red- severe deficiency; orange – mild deficiency; green – sufficient supply. A: Scientific Advisory Committee on Nutrition; Netherlands. B: Institute of Medicine; Australia-New Zealand; Nordic and Deutschland (Germany), Austria and Confederation Helvetica (Switzerland) countries; American Academy of Pediatrics. C: Endocrine Society; International Osteoporosis Foundation; American Geriatrics Society. D: Vitamin D Council and a 'few experts'. Adapted from (1).

In the data analysis the vitamin D levels were evaluated as a continuous variable (ng/ml) and as a categorical variable, following the Endocrine Society guideline: Deficient: < 20 ng/ml; Insufficient: [20, 30[ng/ml; Sufficient: >= 30 ng/ml.

The following table presents the World Health Organization (WHO) clinical progression scale that was used to define the patient disease severity. The data analysis was performed considering the following values for the variable COVID-19 disease severity:

- {4, 5, 6, 7, 8, 9, 10} (ordinal) - there are no uninfected or mild disease cases in this study.
- {Moderate disease, Severe disease, Dead} (categorical)
- {Survived, Dead} (binomial)

Table ii: WHO clinical progression scale for patient disease severity. Adapted from (4).

**Patient State**	**Descriptor**	**Score**
Uninfected	Uninfected, no viral RNA detected	0
Ambulatory mild disease	Asymptomatic; viral RNA detected	1
	Symptomatic; independent	2
	Symptomatic; assistance needed	3
Hospitalised; moderate disease	Hospitalised; no oxygen therapy	4
	Hospitalised; oxygen by mask or nasal prongs	5
Hospitalised: severe disease	Hospitalised; no oxygen by NIV or high flow	6
	Intubation and mechanical ventilation, $pO_2/FiO_2 \geq 150$ or $SpO_2/FiO_2 \geq 200$	7
	Mechanical ventilation $pO_2/FiO_2 <150$ ($SpO_2/FiO_2<200$) or vasopressors	8
	Mechanical ventilation $pO_2/FiO_2<150$ and vasopressors, dialysis, or ECMO	9
Dead	Dead	10

Genetic panel

Table iii describes the genes list and genetic variants that have been tested for each patient. Each gene is described with the following information: gene name, polymorphism RS code, information about the encoded protein, the impact allele for the decreased 25(OH)D, the variant type.

Table iii: Genetic parameters.

Vitamin D Pathway		Encoded protein	Effect allele for decreased 25(OH)D	Variant type
CYP2R1	rs10741657	Vitamin D 25-hydroxylase	G (major)	5' UTR
CYP2R1	rs12794714		A (minor)	Synonymous (NP_078790.2:p.Ser59=)
CYP2R1	rs7116978		C (major)	Intron Variant
GC	rs2282679	Vitamin D-binding protein	G (minor)	Intron Variant
GC	rs1155563		C (minor)	Intron Variant
GC	rs7041		A (minor)	Missense (NP_001191236.1:p.Asp451Glu)
DHCR7	rs12785878	7-Dehydrocholesterol Reductase	G (minor)	Downstream (NADSYN1 : Intron Variant)
DHCR7	rs12800438		G (minor)	Downstream (NADSYN1 : Intron Variant)
DHCR7	rs4944957		A (minor)	Downstream (NADSYN1 : Intron Variant)
CYP24A1	rs6013897	Vitamin D(3) 24-Hydroxylase	A (minor)	Upstream (intergenic)
CYP24A1	rs17216707		C (minor)	Upstream (intergenic)
CYP24A1	rs6127099		T (minor)	Upstream (intergenic)
AMDHD1	rs10745742	Amidohydrolase Domain Containing 1	C (major)	Intron Variant
SEC23A	rs8018720	Protein Transport Protein Sec23A	C (major)	Missense (NP_006355.2:p.Leu211Val (G)) Missense (NP_006355.2:p.Leu211Ile (A))
VDR	rs7975232	Vitamin D receptor	NA	Intron Variant
VDR	rs1544410		NA	Intron Variant
VDR	rs2228570		NA	Missense (NP_001017535.1:p.Met1Thr)
VDR	rs731236		NA	Synonymous (NP_001017535.1:p.Ile352=)

Clinical and phenotypic parameters stored at the e-CRF

Table iv describes the e-CRF main statistics. Each clinical and phenotypic parameters and corresponding information is presented in Table v.

Clinical history, genotypic and phenotypic data, stored at the e-CRF, was collected and managed using REDCap electronic data capture tools hosted by BioData.pt (https://biodata.pt/), the Portuguese distributed infrastructure for biological data, at INESC-ID research institute. REDCap (Research Electronic Data Capture) is a secure, web-based software platform designed to support data capture for research studies, providing 1) an intuitive interface for validated data capture; 2) audit trails for tracking data manipulation and export procedures; 3) automated export procedures for seamless data downloads to common statistical packages; and 4) procedures for data integration and interoperability with external sources (5, 6). All datasets are pseudo-anonymous and only one of them has a key that connects to the patient.

Table iv: e-CRF data description: main statistics.

# patients	517
# clinical parameters	91
# genetic parameters	18
# complete records	491

Table v: Clinical and phenotypic parameters.

Section	Variable	Variable Type
**Patient identification**	Review of inclusion and exclusion criteria	Nominal qualitative variables
**Clinical and Demographics**	Age	Discrete quantitative
	Weight	Continuous quantitative
	Height	Continuous quantitative
	BMI	Continuous quantitative
	Sex	Nominal qualitative
	Symptoms	Nominal qualitative
	Co-morbidities	Nominal qualitative
**Hospitalization**	Medication	Nominal qualitative variables
	Admission Analysis	Nominal quantitative variables
**Outcomes**	Transferred to the Intensive Care Unit during hospitalization?	Nominal qualitative variables
	Oxygen by mask or nasal prongs	
	Oxygen by NIV or high-flow	
	Intubation and mechanical ventilation with pO2/FiO2 >= 150 or SpO2/FiO2 >= 200	
	Mechanical ventilation with pO2/FiO2 < 150 or SpO2/FiO2 < 200, or use of vasopressors	
	Mechanical ventilation with pO2/FiO2 < 150 or SpO2/FiO2 < 200, and use of vasopressors, dialysis or ECMO	
	Dead	
	EAM	
	TEP	
	Stroke	
	Discharged up to 60 days after admission?	
	Length of hospitalization	Continuous quantitative

Polygenic Risk Score

A Polygenic Risk Score (PRS) is an estimate of an individual's genetic liability to a trait or disease, calculated according to their genotype profile and relevant GWAS data.

For a set of genetic variants x, with weights β,

(1) $x = \{x_1, \ldots, x_n\}$
(2) $\beta = \{\beta_1, \ldots, \beta_n\}$

Given an impact function g(x) that returns an impact value for a certain genetic variant x,
(3)

$$g(x) = \begin{cases} 1 & \text{if } x \text{ is impact genotype} \\ 0.5 & \text{if } x \text{ is heterozygous} \\ 0 & \text{otherwise} \end{cases}$$

The PRS is calculated by the following function $f_\beta(x)$,

(4) $$f_\beta(x) = \frac{\sum_{i=1}^{n} \beta_i \cdot g(x_i)}{\sum_{i=1}^{n} \beta_i}$$

For this project, the considered genetic variants and the corresponding impact alleles were selected from GWAS studies conducted in cohorts ranging from 33 996 to 443 374 European individuals showing reproducible genomic hits associated with variation in serum 25(OH)D (2, 3).

The following table describes the values used for the parameter β, for six polymorphisms. These values, which represent the impact of each polymorphism in the model, were obtained simultaneously by a GWAS study with 79,366 individuals with European ancestry (2).

Table vi: Parameters used in the PRS.

Gene	rsID	Effect allele for decreased 25(OH)D	β	p-value
CYP2R1	rs10741657	G (major)	-0.031	2.1e-46
GC	rs2282679	G (minor)	-0.089	4.7e-343
DHCR7	rs12785878	G (minor)	-0.036	3.8e-62
CYP24A1	rs17216707	C (minor)	-0.026	8.1e-23
AMDHD1	rs10745742	C (major)	-0.017	1.9e-14
SEC23A	rs8018720	C (major)	-0.017	4.7e-9

The PRSs did not model other genetic variants that have been tested, since their impact has not been obtained by the same GWAS studies, which could introduce a bias in its relative impact. Simulations have been performed considering the impact of *VDR* gene polymorphisms but no association improvement with COVID-19 severity was observed.

Methodological approach

Regarding the methodological approach, the following steps were undertaken:

1. *Data cleaning and validation:* all variables were analyzed for outliers and missing values. Some discrepancies, such as different units of measure and data entry errors, were identified and fixed. No imputation was made. Regarding data transformation, both disease severity and vitamin D levels were categorized in different levels, and the genetic variants were aggregated in PRSs.

2. *Descriptive analysis:* a complete, graphical descriptive analysis of the data was created for all variables of interest as univariate analysis. Data are presented as numbers or percentages for categorical variables, while continuous variables are shown as mean and standard deviation, and median and interquartile range (25th percentile - 75th percentile).

3. *Analysis of data distribution:* This step provides a clear understanding of what is the underlying distribution that the analyzed parameters follow in the dataset. Statistical normality testing is relevant in order to set up the category of statistical methods (parametric or non-parametric) used in further analysis. The data normality was accessed using Shapiro-Wilk test and D'Agostino Pearson's test. Results showed that most parameters do not follow a normal distribution, thus for further analysis it was considered only non-parametric statistical tests that do not assume any particular data distribution.

4. *Identification of the vitamin D polymorphisms as risk biomarkers:* This step focused on finding differences in genetic variants in vitamin D-related genes between COVID-19 patients with different degrees of disease severity. Several statistical tests were used (following the same assumptions in the former topic), namely Mann-Whitney and Kruskal-Wallis Tests. Spearman rank correlation coefficient was also calculated.

Four PRSs have been computed, focused on the vitamin D metabolism, transport and degradation pathways, based on an additive weighted model, having values in the interval [0, 1]. In this interval, 0 corresponds to a lower risk of having low vitamin D levels due to genetics, and 1 corresponds to a higher risk of having low vitamin D levels due to genetics (see supplemental material for details about the PRSs). The four different scores considered the contribution of the following genetic variants.

(1) Synthesis score = *DHCR7* RS12785878 + *CYP2R1* RS10741657

(2) Metabolism score = *GC* RS 2282679 + *CYP24A1* RS17216707

(3) Pathway score = *DHCR7* RS12785878 + *CYP2R1* RS10741657 + *GC* RS 2282679 + *CYP24A1* RS17216707

(4) Vitamin D total score = *DHCR7* RS12785878 + *CYP2R1* RS10741657 + *GC* RS 2282679 + *CYP24A1* RS17216707 + *AMDHD1* RS10745742 + *SEC23A* RS8018720

5. *Analysis of the correlation between hypovitaminosis D and the disease severity:* This step focused on finding differences in vitamin D blood levels between COVID-19 patients with different degrees of disease severity. Different statistical tests were employed, namely Mann-Whitney and Kruskal-Wallis Tests, depending on the type of categorization under analysis. Spearman rank correlation coefficient was also calculated in order to analyze not only an eventual association but also to quantify it and observe its direction.

6. *Genotypes frequency comparison:* For this comparison the 1000 Genomes (https://www.ensembl.org/index.html) and the HeartGenetics's research database with more than 8,000 Portuguese individuals were used.

Concerning the different statistical tests performed, a p-value < 0.05 was considered statistically significant.

References

(1) Holick MF, et al. Evaluation, treatment, and prevention of vitamin D deficiency: an Endocrine Society clinical practice guideline. *J Clin Endocrinol Metab*. 2011; 96(7):1911-1930. https://doi.org/10.1210/jc.2011-0385

(2) Jiang P, et al. Genome-wide association study in 79,366 European-ancestry individuals informs the genetic architecture of 25-hydroxyvitamin D levels. *Nat Commun*. 2018; 9:260. https://doi.org/10.1038/s41467-017-02662-2

(3) Manousaki D, et al. Genome-wide Association Study for Vitamin D Levels Reveals 69 Independent Loci. *Am. J. Hum. Genet*. 2020; 106(3):327-337 https://doi.org/10.1016/j.ajhg.2020.01.017

(4) Marshall, JC, et al. A minimal common outcome measure set for COVID-19 clinical research. *The Lancet Infectious Diseases.* 2020; 20(8):E192-E197 https://doi.org/10.1016/S1473-3099(20)30483-7

(5) Harris PA, et al. The REDCap consortium: Building an international community of software platform partners. *J Biomed Inform*. 2019; 95:103-208. https://doi.org/10.1016/j.jbi.2019.103208

(6) Harris PA, et al., Research electronic data capture (REDCap)--a metadata-driven methodology and workflow process for providing translational research informatics support. *J Biomed Inform*. 2009; 42(2):377-381. https://doi.org/10.1016/j.jbi.2008.08.010
